# Short-Term Outcomes of Patients With COVID-19 Undergoing Invasive Mechanical Ventilation: A Retrospective Observational Study From Wuhan, China

**DOI:** 10.3389/fmed.2020.571542

**Published:** 2020-09-29

**Authors:** Shuai Zhao, Yun Lin, Cheng Zhou, Li Wang, Xueyin Chen, Sean P. Clifford, Ozan Akca, Jiapeng Huang, Xiangdong Chen

**Affiliations:** ^1^Department of Anesthesiology, Tongji Medical College, Union Hospital, Huazhong University of Science and Technology, Wuhan, China; ^2^Laboratory of Anesthesia and Critical Care Medicine, Department of Anesthesiology, Translational Neuroscience Center, West China Hospital of Sichuan University, Chengdu, China; ^3^Department of Anesthesiology and Perioperative Medicine, University of Louisville, Louisville, KY, United States

**Keywords:** COVID-19, SARS-CoV-2, invasive mechanical ventilation, critically ill patients, outcomes

## Abstract

**Background:** COVID-19 has spread rapidly worldwide. Many patients require mechanical ventilation. The goal of this study was to investigate the clinical course and outcomes of patients with COVID-19 undergoing mechanical ventilation and identify factors associated with death.

**Methods:** Eighty-three consecutive critically ill patients with confirmed COVID-19 undergoing invasive mechanical ventilation were included in this retrospective, single-center, observational study from January 31 to March 15, 2020. Demographic, clinical, laboratory, radiological, and mechanical ventilation data were collected and analyzed. The primary outcome was 28-day mortality after endotracheal intubation. The secondary outcomes included the incidences of SARS-CoV-2-related cardiac, liver, and kidney injury.

**Results:** Seventy-four out of 83 (89.2%) patients achieved oxygen saturation above 93% after intubation. Forty-nine out of 83 (59%) patients died and 34 (41%) patients survived after 28 days of observation. Multivariable regression showed increasing odds of death associated with cardiac injury (odds ratio 15.60, 95% CI 4.20–74.43), liver injury (5.40, 1.46–23.56), and kidney injury (8.39, 1.63–61.41), and decreasing odds of death associated with the higher PaO_2_/FiO_2_ ratio before intubation (0.97, 0.95–0.99). PaO_2_/FiO_2_ ratio before intubation demonstrated a positive linear correlation with platelet count (*r* = 0.424, *P* = 0.001), and negative linear correlation with troponin I (*r* = −0.395, *P* = 0.008).

**Conclusions:** Cardiac, liver, and kidney injury may be associated with death for critically ill patients with COVID-19 undergoing invasive mechanical ventilation. The severity of pre-intubation hypoxia may be associated with a poorer outcome of patients with COVID-19 undergoing invasive mechanical ventilation. Larger, multi-institutional, prospective studies should be conducted to confirm these preliminary results.

## Introduction

An ongoing outbreak of SARS-CoV-2 has spread rapidly worldwide (1–4). The epidemiological findings, clinical presentation, and clinical outcomes of patients with COVID-19 have been reported (2, 5–8). However, the clinical course and outcomes of patients with COVID-19 undergoing invasive mechanical ventilation is still not clear.

Confirmed COVID-19 patients with comorbidities can rapidly develop acute respiratory distress syndrome and require intensive care unit admission as well as invasive/non-invasive mechanical ventilation (6, 7, 9). The overall mortality rate exceeds nearly 60% in critically ill COVID-19 patients from the Jin Yin-tan hospital study (7). No vaccine or specific effect medicines for COVID-19 has yet been shown to be effective, so invasive mechanical ventilation via endotracheal intubation is particularly important for severe cases to slow progression and reduce mortality (6, 7, 10). As many critically ill patients require invasive mechanical ventilation, it is critical to gain a deeper understanding of the association between mechanical ventilation and its subsequent clinical outcomes. In addition, it has previously been observed that multi-organ injury, such as lung, heart, kidney, liver injury is a common condition among hospitalized patients with COVID-19, and it may associate with higher risk of in-hospital mortality (8, 11–13). However, the progression of organ injury and outcomes of critically ill patients undergoing mechanical ventilation remain poorly defined. Identifying or more promptly treating patients undergoing mechanical ventilation is crucial to decrease the mortality rate.

Therefore, in this retrospective study, we aimed to investigate the clinical course and outcomes of patients with COVID-19 undergoing mechanical ventilation and identify factors associated with death.

## Materials and Methods

### Study Design and Participants

For this single-center, retrospective, and observational study, we included 83 consecutive patients with confirmed COVID-19 undergoing invasive mechanical ventilation at the West Campus of Union Hospital from January 31 to February 15, 2020. This hospital is one of the main designated tertiary care centers for severe cases of COVID-19. We retrospectively collected and analyzed the patients with laboratory-confirmed COVID-19 according to World Health Organization (WHO) interim guidance (9). No sample size calculation was performed a priori due to the exploratory nature of this study, and thus we included all possible critically ill COVID-19 patients in our investigation.

This study was approved by the institutional ethics committee of Union Hospital, Tongji Medical College, Huazhong University of Science and Technology (No. 20200097). Written informed consent was waived by the hospital's ethics commission. This manuscript adheres to the applicable CONSORT guidelines.

### Data Collection

The clinical electronic medical records, nursing records, laboratory findings, and chest x-rays of all patients were analyzed. Clinical characteristics, laboratory findings, radiological data, as well as treatment and outcomes data were collected with standardized data collection forms (modified from the standardized International Severe Acute Respiratory and Emerging Infection Consortium case report forms) (14). Data were independently verified to ensure accuracy by two investigators. The researchers also directly communicated with involved health-care providers and patient family members to minimize data loss, and ensure the accuracy and reliability of the data. We collected data including age, sex, chronic medical histories (hypertension, cardiovascular disease, chronic pulmonary disease, chronic neurological disorder, chronic kidney disease, diabetes), vital signs (heart rate, blood pressure, SpO_2_, PaO_2_), laboratory findings, chest CT scans, electrocardiogram (ECG), and information on oxygenation status (PaO_2_/FiO_2_ ratio [P/F] and SpO_2_ before and after intubation). Vital signs and laboratory parameters were collected on admission into hospital (ADM-) and continuously tracked daily from pre-intubation (PRE-) until 7 days after intubation (POST-1 to POST-7). The partial pressure of arterial oxygen (PaO_2_) was measured by arterial blood gas analysis. The P/F was calculated using the formula PaO_2_/FiO_2_ (mmHg).

### Procedures of Intubation and Mechanical Ventilation

Endotracheal intubation was performed by experienced anesthesiologists using airborne precautions according to the interim guidance recommended by the World Health Organization (WHO): patients receiving high-flow nasal oxygen should be in a monitored setting and cared for by experienced personnel capable of endotracheal intubation in case the patient acutely deteriorates or does not improve after a short trial (about 1 h) (9). All study patients were intubated orally using a video laryngoscope. Vital signs including the patient's SpO_2_, blood pressure, heart rate, and breathing status were closely observed and recorded after intubation. A protective ventilation strategy with small tidal volumes (e.g., 4–6 ml.kg^−1^ ideal body weight), low inspiratory pressures (Pplat < 30 cmH_2_O) and optimal PEEP [by ARDSNet (15)] was used to reduce ventilator-induced lung injury. Unfortunately, the exact data on mechanical ventilation were not recorded and the compliance rate with the protective mechanical ventilation were not known.

### Outcome Measures

The primary outcome was 28-day mortality after endotracheal intubation with the final date of follow-up being March 15, 2020. Secondary outcomes included the incidences of SARS-CoV-2-related cardiac, liver, and kidney injury. Cardiac injury was defined as the serum levels of troponin I (TnI) above the upper limit of the reference range (>26 ng.L^−1^) or new ST segmental and T-wave changes or pathologic Q-waves found on ECG (16). Liver injury was diagnosed if the serum levels of aspartate transaminase (AST), alanine transaminase (ALT), and AST/ALT were above the upper limit of the reference range (ALT > 40 U/L; AST > 40 U/L; AST/ALT > 1) (17). Kidney injury was diagnosed if the serum levels of creatinine and blood urea nitrogen (BUN) were above the upper limit of the reference range (creatinine > 133 μmol.L^−1^; BUN > 8.2 mmol.L^−1^) (18). All laboratory findings mentioned above were provided by the laboratory of West Campus of Union Hospital.

The Sequential Organ Failure Assessment (SOFA) was calculated on admission and every 24 h until the final date of follow-up. The worst values for each parameter in the 24-h period were used in scoring tabulation. The daily SOFA score was calculated for each patient on the basis of six organ systems: cardiovascular, respiratory, renal, hepatic, coagulation, and neurologic systems (scores for each system range from 0 to 4, with higher scores indicating more severe organ-system dysfunction; maximum score, 24).

### Statistical Analysis

Continuous data were presented as mean (SD) or median (IQR), and categorical data were presented as number (%). Means for continuous data were compared using independent group *t*-tests when the data were normally distributed; otherwise, the Mann-Whitney *U*-test was used. Proportions for categorical variables were compared using the Chi-square (and Fisher's exact) test. Survival analysis was performed using Kaplan-Meier survival curves. Univariable and multivariable logistic regression models were used to explore the risk factors associated with death for patients with COVID-19. Considering the total number of deaths (*n* = 49) in this study and to avoid overfitting in the model, five variables (age, P/F, cardiac injury, liver injury, and kidney injury) were chosen for multivariable analysis on the basis of previous findings and clinical constraints. The correlation coefficient was calculated by Pearson's correlation analysis. A two-sided α of <0.05 was considered statistically significant. All statistical analysis was performed with GraphPad Prism version 8 (Graph-Pad Software Inc., San Diego, CA, U.S.) and SPSS software (version 25 for Mac; IBM, New York, USA).

## Results

**Figure 1:**
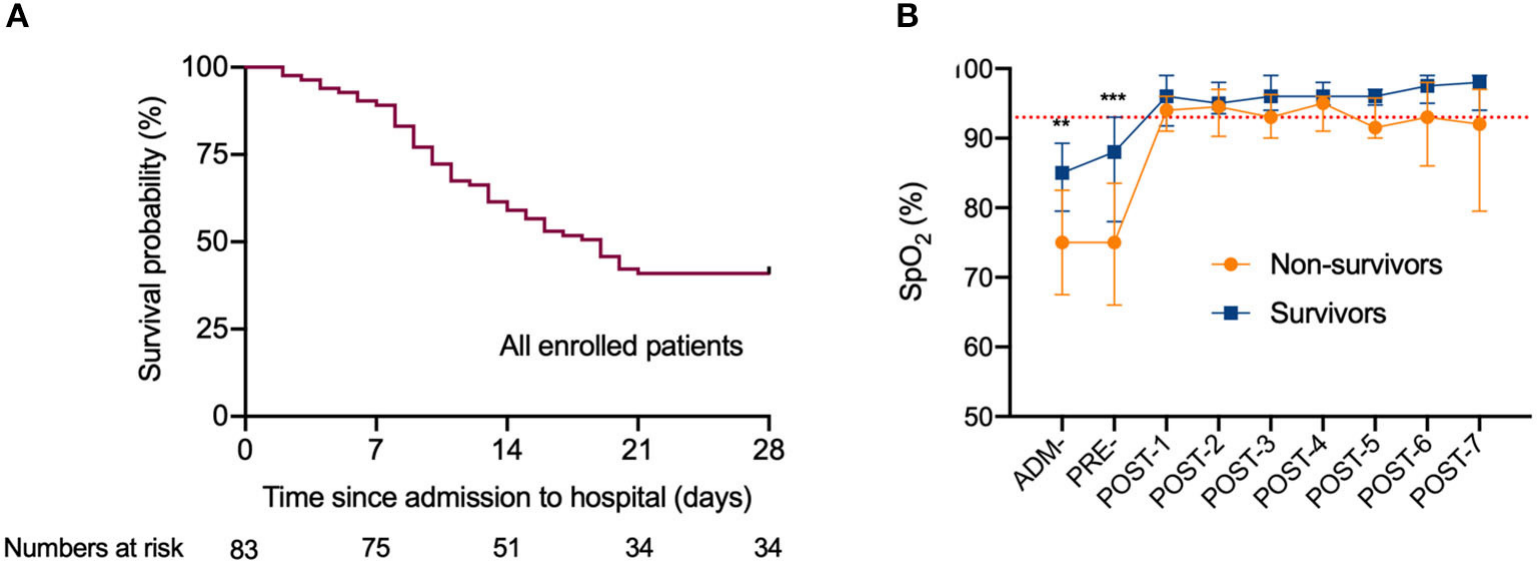
The survival curve of critically ill patients with COVID-19 undergoing invasive mechanical ventilation, and dynamic changes of the oxygen saturation during hospitalization. **(A)** The survival curve of all enrolled patients with COVID-19 undergoing invasive mechanical ventilation. **(B)** Dynamic changes of the oxygen saturation during hospitalization. The dotted lines in red show the SpO_2_ at 93%.Data are median (IQR). *Indicate difference between the survivors and non-survivors by *t*-tests or Mann-Whitney *U*-test. Significant differences are expressed as follows: * < 0.05, ** < 0.01, *** < 0.001. SpO_2_, oxygen saturation; ADM-, admission to hospital; PRE-, pre-intubation; POST-1 to POST-7, post-intubation day 1 to post-intubation day 7.

We observed 83 critically ill patients with confirmed COVID-19 undergoing invasive mechanical ventilation. In the cohort of 83 consecutive patients, the median (IQR) age was 65 (60–71) years, and 58 (69.9%) patients were men (Table 1). Sixty-four (46.4%) patients had one or more chronic medical illness including: hypertension (42 [50.6%]), cardiovascular disease (13 [15.7%]), chronic pulmonary disease (7 [8.4%]), chronic neurological disorder (3 [3.6%]), chronic liver disease (5 [6.0%]), chronic kidney disease (4 [4.8%]), and diabetes (30 [36.1%]) (Table 1). Eighty-three (100%) patients showed bilateral distribution of patchy shadows or ground glass opacity. The median (IQR) duration from admission to intubation for total patients was 6 (4–11). By March 15, 2020, 23 (27.7%) patients remained intubated in the hospital, 11 (13.3%) patients had been successfully extubated, and 49 (59.0%) patients died (Table 1). The median survival time of total patients was 19 (IQR 10–28) days (Figure 1).

**Table 1:** Baseline characteristics, laboratory results, chest CT findings, and clinical outcomes of survivors and non-survivors with COVID-19.

	**Total patients (*N =* 83)**	**Survivors (*N =* 34)**	**Non-survivors (*N =* 49)**	***P*-value**
**Baseline characteristics**				
**Age, years**				
Mean (SD)	64 (11.0)	61 (10.6)	66 (10.9)	0.039
Median (IQR)	65 (60–71)	62 (55–68.8)	68 (63–71)	
**Sex, No. (%)**				
Female	25 (30.1)	9 (26.5)	16 (32.7)	0.546
Male	58 (69.9)	25 (73.5)	33 (67.3)	
**Chronic medical illness, No. (%)**				
Hypertension	42 (50.6)	12 (35.3)	30 (61.2)	0.020
Cardiovascular disease	13 (15.7)	4 (11.8)	9 (18.4)	0.416
Chronic pulmonary disease	7 (8.4)	1 (2.9)	6 (12.2)	0.231
Chronic neurological disorder	3 (3.6)	1 (2.9)	2 (4.1)	>0.999
Chronic liver disease	5 (6.0)	1 (2.9)	4 (8.2)	0.644
Chronic kidney disease	4 (4.8)	2 (5.9)	2 (4.1)	0.706
Diabetes	30 (36.1)	10 (29.4)	20 (40.8)	0.288
**Vital signs at pre-intubation, median (IQR)**				
Heart rate, bpm	99 (84.3–119.3)	92 (79.3–116.8)	100 (88.3–122.3)	0.564
Systolic pressure, mmHg	132 (120.0–153.5)	128 (116.0–147.5)	137 (123–157)	0.684
Diastolic pressure, mmHg	80 (67.0–91.5)	73 (66.8–92.3)	85 (69.5–90.5)	0.979
MAP, mmHg	93 (86–115)	91 (83–102)	98 (86–113)	0.735
SpO_2_	82 (71.5–89.3)	88 (78.0–92.5)	79 (66.5–84.0)	0.001
PaO_2_, mmHg	53.1 (45.6–59.5)	59 (52.5–64.3)	49 (42.6–55.4)	<0.001
P/F at pre-intubation	121 (103.6–135.2)	134 (119.4–146.1)	112 (96.8–125.8)	<0.001
**P/F at pre-intubation, No. (%)**				
P/F >150 mmHg	10 (12.0)	7 (20.6)	3 (6.1)	0.515
100 ≤ P/F ≤ 150 mmHg	55 (66.3)	26 (76.5)	29 (59.2)	0.156
P/F <100 mmHg	18 (21.7)	1 (2.9)	17 (34.7)	<0.001
**Laboratory results at pre-intubation, median (IQR)**				
Leucocytes count, × 10^9^.L^−1^	12.46 (9.15–16.65)	12.38 (8.68–15.36)	12.53 (9.50–17.22)	0.982
Lymphocyte count, × 10^9^.L^−1^	0.54 (0.42–0.71)	0.67 (0.45–0.83)	0.49 (0.35–0.60)	0.107
Neutrophil count, × 10^9^.L^−1^	9.73 (7.82–16.57)	9.60 (7.28–15.04)	9.95 (8.47–17.03)	0.362
Platelet count, × 10^9^.L^−1^	147 (98–197)	134 (90.50–185)	120 (69.75–167)	0.021
ALT, U.L^−1^	42 (26–68)	53 (38–93.50)	30 (20–54)	0.425
AST, U.L^−1^	41 (30–68)	47 (28.50–68.50)	38 (31.25–65.50)	0.926
AST/ALT	0.99 (0.65–1.31)	0.82 (0.60–1.03)	1.14 (0.83–1.450)	0.011
BUN, mmol.L^−1^	8.73 (5.78–11.51)	7.55 (4.92–8.97)	9,35 (6.68–12.50)	0.047
Creatinine, μmol.L^−1^	65.9 (54.80–88.50)	62.9 (52.30–76.93)	69.6 (56.60–89.80)	0.338
BNP, pg.ml^−1^	86.34 (48.35–138.08)	63.7 (45.2–89.23)	114.6 (64.28–185.50)	0.051
TnI, ng.L^−1^	34.78 (19.96–77.68)	24.4 (16.25–51.87)	52.65 (23.48–87.93)	0.362
CRP, mg.L^−1^	81.9 (54.6–131.0)	87.2 (46.5–131.0)	81.4 (61.7–130.5)	0.750
**Chest CT findings, No. (%)**				
Bilateral distribution of patchy shadows or ground glass opacity	83 (100)	34 (100)	49 (100)	
**Duration from admission to intubation, median (IQR)**	6 (4–11)	8 (4–12)	6 (3–10)	0.088
**Complications, No. (%)**				
Cardiac injury	37 (44.6)	8 (23.5)	29 (59.2)	0.001
Liver injury	36 (43.4)	8 (23.5)	28 (57.1)	0.002
Kidney injury	25 (30.1)	3 (8.8)	22 (44.9)	<0.001
**SOFA score at post-intubation day 3, median (IQR)**	5 (4–6)	4 (4–4.8)	6 (5–7)	<0.001
**Clinical outcome, No. (%)**				
Hospitalization with extubation	11 (13.3)	11 (32.4)	0	
Hospitalization with intubation	23 (27.7)	23 (67.6)	0	
Died	49 (59.0)	0	49 (59.0)	
**Survival time, median (IQR)**	19 (10–28)	28 (28–28)	11 (8–16)	<0.001

*Data are mean (SD), median (IQR) and No. (%). P-values indicate differences between survivors and non-survivors. P < 0.05 was considered statistically significant. IQR, interquartile range; P/F, PaO_2_/FiO_2_ ratio; MAP, mean arterial pressure; SpO_2_, oxygen saturation; PaO_2_, partial pressure of oxygen; ALT, alanine transaminase; AST, aspartate transaminase; BUN, blood urea nitrogen; BNP, brain natriuretic peptide; TnI, troponin I; CRP, C reactive protein; SOFA, Sequential Organ Failure Assessment*.

**Figure 2:**
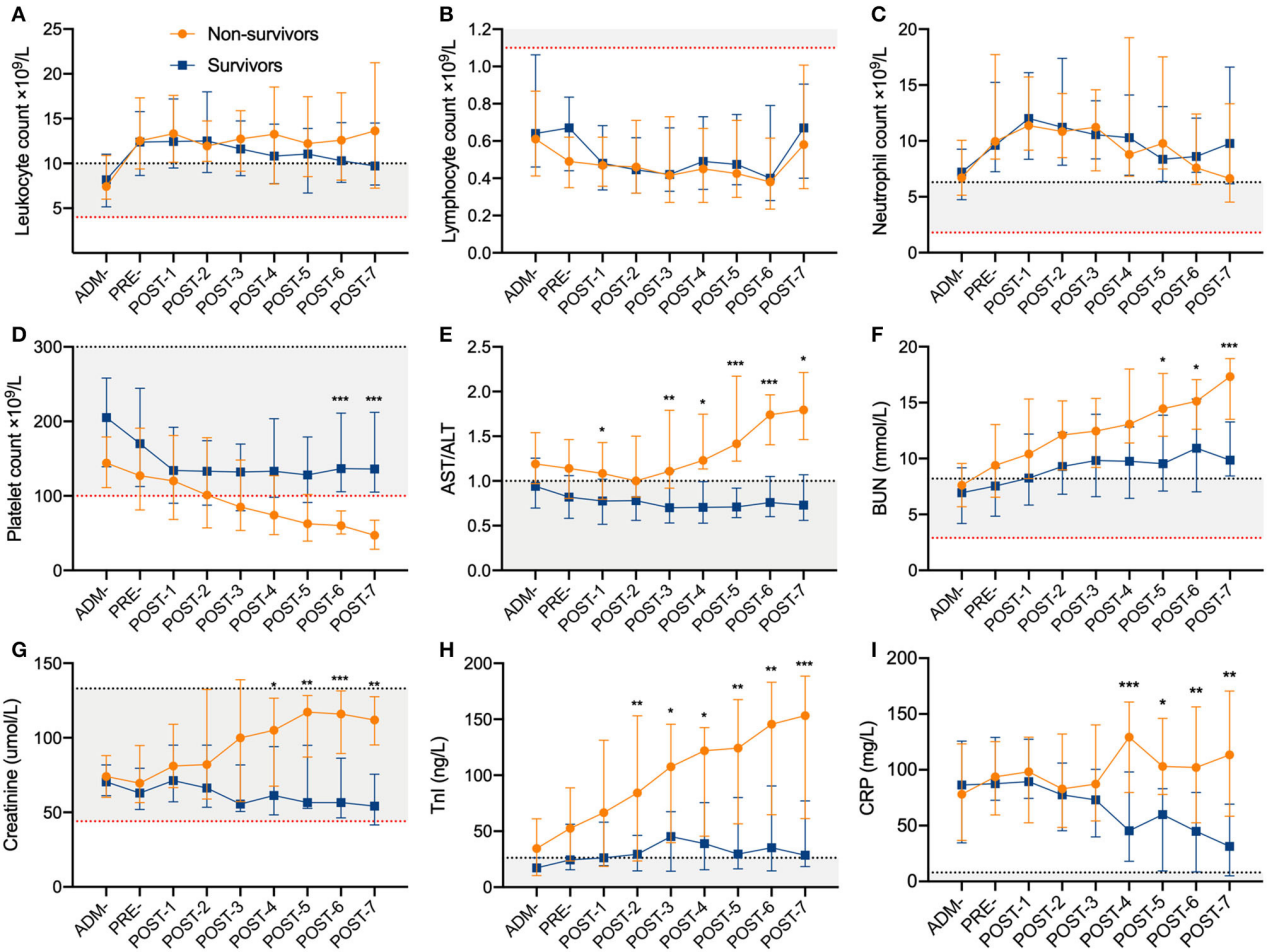
Dynamic changes of laboratory parameters during hospitalization for survivors and non-survivors. **(A)** Leukocyte count, **(B)** Lymphocyte count, **(C)** Neutrophil count, **(D)** Platelet count, **(E)** AST/ALT, **(F)** BUN, **(G)** Creatinine, **(H)** TnI, **(I)** CRP. The dotted lines in red/black show the lower/upper normal limit of each parameter, and the shaded areas represent the normal reference range of each parameter. Data are median (IQR). *Indicate difference between the survivors and non-survivors by *t* tests or Mann-Whitney *U* test. Significant differences are expressed as follows: * < 0.05, ** < 0.01, *** < 0.001. IQR, interquartile range; ALT, alanine transaminase; AST, aspartate transaminase; BUN, blood urea nitrogen; TnI, troponin I; CRP, C reactive protein; ADM-, admission to hospital; PRE-, pre-intubation; POST-1 to POST-7, post-intubation day 1 to post-intubation day 7.

We divided all patients into survivors (*n* = 34) and non-survivors (*n* = 49) (Table 1). Regarding the primary outcome, 49 (59.0%) of 83 patients died within 28 days of observation even though 74 (89.2%) patients achieved oxygen saturation above 93% after invasive mechanical ventilation was initiated (Figure 1). Prior to intubation, the median (IQR) SpO_2_ with O_2_ supplement were 79 (66.5–84.0) vs. 88 (78.0–92.5), *P* =0.001 for non-survivors vs. survivors, and the median (IQR) PaO_2_/FiO_2_ ratio were 112 (96.8–125.8) vs. 134 (119.4–146.1), *P* < 0.001 for non-survivors vs. survivors. Post intubation, SpO_2_ were 94 (91–96) vs. 96 (93–99), *P* = 0.117. The average age of the survivors was younger than the non-survivors (61 vs. 66 years old, *P* = 0.039). No significant difference was found in the duration from admission to intubation between the survivors and non-survivors (*P* = 0.088) (Table 1). At pre-intubation, the differences in levels of leucocytes, lymphocytes, and neutrophils between survivors and non-survivors were not statistically significant, although these values were abnormal in both groups (Figure 2).

Compared with survivors, non-survivors developed higher incidences of SARS-CoV-2-related cardiac (29 [59.2%] vs. 8 [23.5%]; *P* = 0.001), liver (28 [57.1%] vs. 8 [23.5%]; *P* = 0.002), and kidney injury (22 [44.9%] vs. 3 [8.8%]; *P* < 0.001) (Table 1). It is worth noting that non-survivors showed lower blood pressure, higher level of CRP (C reactive protein), and progressive thrombocytopenia, cardiac injury, liver injury and kidney injury from about the 3rd day post intubation (Figure 2). Non-survivors showed a higher SOFA score (6 [IQR 5–7] vs. 4 [4–4.8]; *P* < 0.001) at post-intubation day 3 (Table 1).

In univariable analysis, odds ratios of death (49 non-survivors vs. 34 survivors) was higher in patients with lower P/F before intubation, AST/ALT >1, BNP ≥100 pg/ml and TnI ≥26.2 ng/L. Cardiac, liver, and kidney injury were also associated with death for critically ill patients with COVID-19 undergoing invasive mechanical ventilation (Table 2). When including five variables in the multivariable logistic regression model, we found that P/F pre-intubation, cardiac, liver, and kidney injury were associated with death for critically ill patients with COVID-19 undergoing invasive mechanical ventilation (Table 2).

**Table 2:** Risk factors associated with death for patients with COVID-19 undergoing invasive mechanical ventilation.

	**Univariable OR (95% CI)**	***P*-value**	**Multivariable OR (95% CI)**	***P*-value**
**Baseline characteristics**				
Age, years	1.05 (1.00–1.10)	0.049	1.03 (0.97–1.09)	0.343
Male sex (vs. female)	0.74 (0.27–1.93)	0.547		
**P/F pre–intubation, mmHg**				
P/F	0.97 (0.95–0.99)	0.002	0.97 (0.95–0.99)	0.006
**Laboratory results at post–intubation day 3, median (IQR)**				
**Platelet count**, **×** **10**^**9**^**.L**^**−1**^				
>100	0.47 (0.15–0.38)	0.175		
<100	2.13 (0.72–6.48)	0.175		
**AST/ALT**				
≥1	5.13 (1.80–15.83)	0.003		
<1	0.19 (0.06–0.56)	0.003		
**BUN, mmol.L**^**−1**^				
≥8.2	2.16 (0.65–7.60)	0.215		
<8.2	0.46 (0.13–1.54)	0.215		
**Creatinine**, **μmol.L**^**−1**^				
≥133	2.86 (0.73–14.29)	0.154		
<133	0.35 (0.70–1.37)	0.154		
**BNP, pg.ml**^**−1**^				
≥100	4.00 (1.28–13.46)	0.019		
<100	0.25 (0.07–0.78)	0.019		
**TnI, ng.L**^**−1**^				
≥26.2	11.00 (3.05–48.37)	<0.001		
<26.2	0.09 (0.02–0.33)	<0.001		
**Complications**				
Cardiac injury	8.04 (2.95–24.95)	<0.001	15.60 (4.20–74.43)	0.001
Liver injury	4.33 (1.69–12.04)	0.003	5.40 (1.46–23.56)	0.016
Kidney injury	8.42 (2.56–38.38)	0.002	8.39 (1.63–61.41)	0.019

*Data are Odds Ratios (95% CI). P < 0.05 was considered statistically significant. P/F, PaO_2_/FiO_2_ ratio; ALT, alanine transaminase; AST, aspartate transaminase; BUN, blood urea nitrogen; BNP, brain natriuretic peptide; TnI, troponin I*.

**Figure 3:**
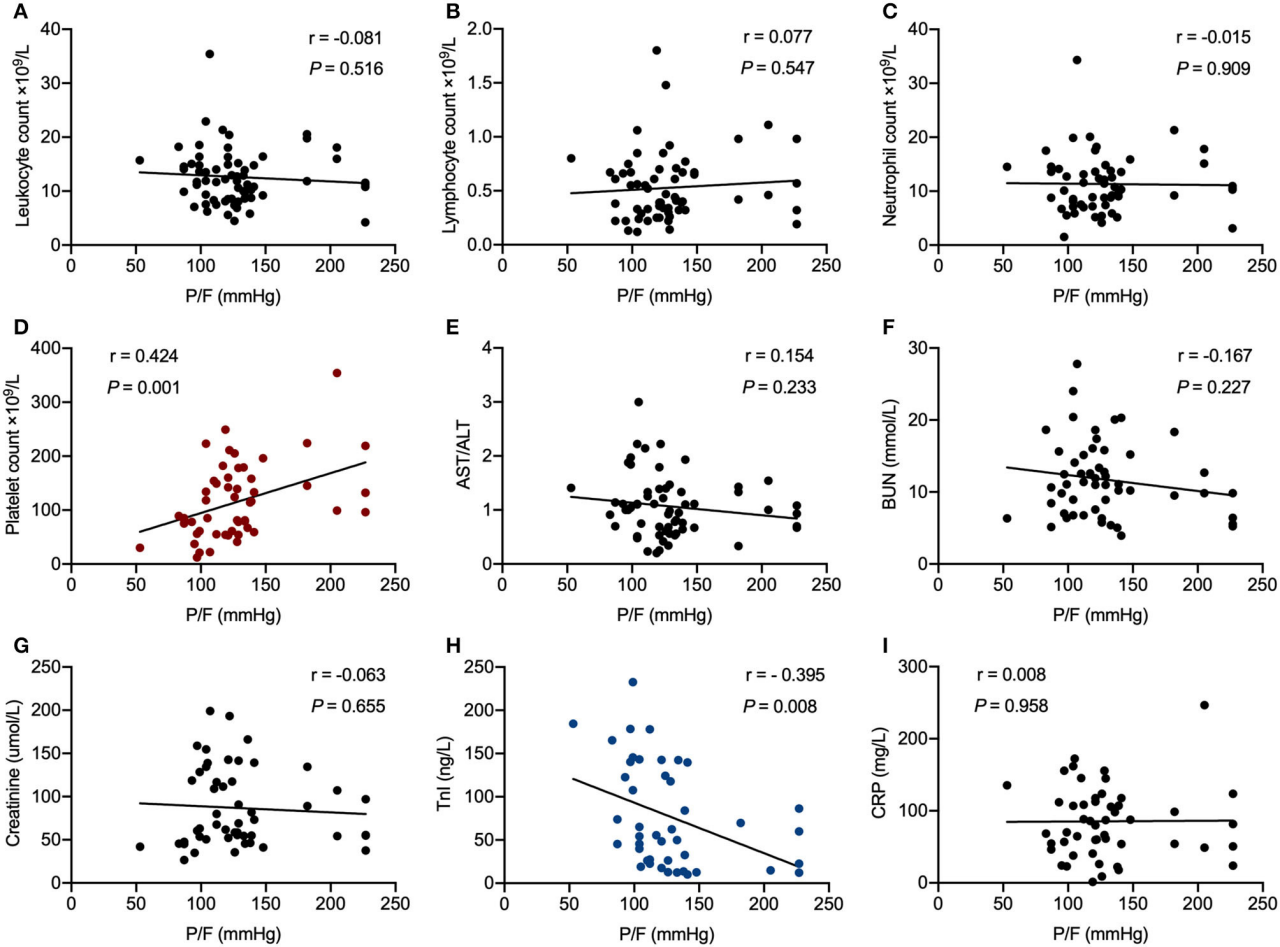
Correlation analysis of PaO_2_/FiO_2_ ratio before intubation with laboratory parameters in critically ill patients with COVID-19 undergoing invasive mechanical ventilation. **(A)** Leukocyte count, **(B)** Lymphocyte count, **(C)** Neutrophil count, **(D)** Platelet count, **(E)** AST/ALT, **(F)** BUN, **(G)** Creatinine, **(H)** TnI, **(I)** CRP. R represents the Pearson's correlation coefficient. P/F, PaO_2_/FiO_2_ ratio; ALT, alanine transaminase; AST, aspartate transaminase; BUN, blood urea nitrogen; TnI, troponin I; CRP, C reactive protein.

In correlation analysis, P/F before intubation in patients with COVID-19 correlated significantly with platelet count (*r* = 0.424, *P* = 0.001) and TnI (*r* = −0.395, *P* = 0.008) (Figure 3).

## Discussion

In this study, we report on the clinical course and outcomes of 83 critically ill patients with confirmed COVID-19 undergoing invasive mechanical ventilation. Forty-nine out of 83 (59%) patients died and 34 (41%) patients survived after 28 days of observation. Cardiac, liver, and kidney injury may be associated with death for critically ill patients with COVID-19 undergoing invasive mechanical ventilation. The severity of pre-intubation hypoxia may be associated with a poorer outcome of patients with COVID-19 undergoing invasive mechanical ventilation.

In accordance with the findings of other investigators, our critically ill patients with COVID-19 demonstrated leukocytosis, lymphopenia, thrombocytopenia, and high neutrophil levels, as well as developed different degrees of organ injury in a short period of time (2, 6, 7, 19, 20). Notably, non-survivors showed marked thrombocytopenia, cardiac injury, liver injury, kidney injury, and higher SOFA scores compared with survivors after intubation, even though all patients demonstrated similar SpO_2_ levels after the initiation of mechanical ventilation. The incidences of cardiac injury (59.2 vs. 28.0%), liver injury (57.1 vs. 28.0%), and kidney injury (44.9 vs. 37.5%) in this investigation were higher compared to the previous study from Jin Yin-tan Hospital (7). Moreover, cardiac, liver, and kidney injury in non-survivors occurred much earlier: organ failure became prominent around the 3rd day after initiation of invasive mechanical ventilation in non-survivors compared with survivors. SOFA scores at post-intubation day 3 were higher in non-survivors than survivors as well. This may be a manifestation of an accelerated disease progression, with earlier detection and intervention in cardiac, liver, and kidney injury might be used to reduce mortality. Our results of multivariable logistic regression model also preliminarily suggested that cardiac, liver, and kidney injury may be associated with death clinically. In particular, the rapid cardiac function deterioration of these patients may expedite death. Taken as a whole, we speculate that multi-organ injury, especially cardiac, liver, and kidney injury, may be the leading potential causes of death rather than the hypoxemia and severe acute lung injury. Even though our results cannot draw a firm association due to the characteristics of retrospective exploratory study with a relatively small sample size, it could potentially provide a clue for further investigation of the value of these predictors in clinical practice.

Multi-organ function damage, including acute lung injury, acute kidney injury, cardiac injury, and liver dysfunction has been widely reported in COVID-19 (2, 6, 7). Diffuse alveolar damage, pulmonary edema with hyaline membrane formation, and hepatocyte steatosis were reported from pathologic examinations of a patient who died from SARS-CoV-2 (21). Previous studies have revealed that the SARS-CoV-2 uses the same cell entry receptor, angiotensin-converting enzyme 2 (ACE2), as SARS-CoV, to invade the human host and cause a primary pneumonia (22). ACE2 is widely expressed in the testis, heart, kidney, small intestine, thyroid, and adipose tissue. In the lungs, liver, colon, bladder, and adrenal gland, ACE2 showed moderate expression levels as well (23). It provides potential cues that SARS-CoV-2 may damage other tissues and organs outside the lungs, such as the heart, liver, and kidney. In addition, non-survivors in our study showed lower blood pressure and higher level of C reactive protein (CRP), these changes likely reflected hypoperfusion of organ, increased inflammation and organ injury. However, no direct evidence of the perfusion in lung, heart, and other organs was available.

In our study, the P/F ratio before intubation demonstrated a significantly positive linear correlation with platelet count, and negative linear correlation with TnI. It is notable that patients developed different outcome even though most patients achieved oxygen saturation above 93% after intubation. This may indicate that the correcting hypoxia with mechanical ventilation seems not associated with a better outcome. The early occurrence and continuous increase of TnI and decrease of PLT predict the poor outcome. Multiple organs are sensitive to hypoxic insult under ARDS, including the brain, heart, lung, and kidney (10). Therefore, we speculate that critically ill patients with poor oxygenation are more vulnerable to myocardial damage especially under the cytokine storm induced by SARS-CoV-2 invasion. It is difficult to reverse this progression even when oxygen saturation remains above 93% following initiation of invasive mechanical ventilation. In addition, fulminant myocarditis induced by SARS-CoV-2 has been reported from Jin Yin-tan Hospital and Tongji Hospital (24). Acute cardiac injury may be related to direct and/or indirect effects of the SARS-CoV-2, hypoxia, shock, severe immune injury, and medications. It might be very difficult to distinguish hypoxia triggered cardiac injury vs. innate cardiac injury from the virus. However, given that cardiac injury is characterized by a rapid progression and a severe state of illness, our findings should alert physicians to pay attention not only to the symptoms of respiratory failure but also to the cardiac injury as well.

Although the P/F ratio before intubation was included in the logistic regression analysis, the conclusions that can be drawn from the current results were limited due to the non-controlled intubation timing (P/F ratio pre-intubation), OR (0.97, range 0.95–0.99) value close to 1 and unknown compliance rate with protective mechanical ventilation strategies. Firstly, given the retrospective study design and limited medical resources at the time of study, the timing of invasive mechanical ventilation was not controlled. Secondly, the OR value [0.97 (0.95–0.99)] for the P/F ratio before intubation was very close to 1, which may imply a statistically significant but not clinically meaningful difference. Thirdly, although protective mechanical ventilation strategy was recommended for all patients, the exact data on mechanical ventilation were not recorded and the compliance rate with the protective mechanical ventilation were not known. Thus, the identified association between the P/F ratio before intubation and mortality should be evaluated with cautions. Investigating the association between the management of invasive mechanical ventilation and clinical outcomes in COVID-19 patients is of great importance clinically (25, 26). Different criteria for initiating invasive mechanical ventilation and different management strategies of mechanical ventilation may both affect the overall outcomes of COVID-19 patients. Further study is warranted in a prospective and larger cohort to confirm.

Our study has several limitations. Firstly, no sample size calculation was performed a priori due to the exploratory nature of this study, and thus we included all possible critically ill COVID-19 patients in our institution. Secondly, this was a retrospective exploratory study with relatively small sample size. The associations observed in the retrospective observational study need further confirmation in larger cohorts and prospective studies. Thirdly, mechanical ventilation protocol was adjusted with changes of patients' situations and detailed mechanical ventilation data (tidal volume, PEEP, driving pressure, etc.) were not available.

To the best of our knowledge, this is the largest retrospective cohort study among patients with COVID-19 undergoing invasive mechanical ventilation. We found that cardiac, liver, and kidney injury may be associated with death for critically ill patients with COVID-19 undergoing invasive mechanical ventilation. The severity of pre-intubation hypoxia may be associated with a poorer outcome of patients with COVID-19 undergoing invasive mechanical ventilation. Larger, multicenter, prospective studies should be conducted to confirm these findings.

## Data Availability Statement

The raw data supporting the conclusions of this article will be made available by the authors, without undue reservation.

## Ethics Statement

The studies involving human participants were reviewed and approved by Union Hospital, Tongji Medical College, Huazhong University of Science and Technology. Written informed consent for participation was not required for this study in accordance with the national legislation and the institutional requirements.

## Author's Note

This author takes responsibility for all aspects of the reliability and freedom from bias of the data presented and their discussed interpretation.

## Author Contributions

XiC: had full access to all of the data in the study, take responsibility for the integrity of the data and the accuracy of the data analysis, concept and design, and obtained funding. SZ, YL, CZ, LW, XuC, SC, JH, and XiC: acquisition, analysis, or interpretation of data. SZ and YL: drafting of the manuscript and statistical analysis. SZ, YL, CZ, SC, OA, JH, and XiC: critical revision of the manuscript for important intellectual content. All authors: contributed to the article and approved the submitted version.

## Conflict of Interest

The authors declare that the research was conducted in the absence of any commercial or financial relationships that could be construed as a potential conflict of interest.
